# Manipulative Surgery

**Published:** 1917-03-31

**Authors:** 


*To the Editor of* The Hospital.

### Manipulative Surgery.

*To the Editor of* The Hospital.

Sir,—You refer to Mr. Barker the bonesetter. May I, in fairness to him, give my experience? Some years ago I became suddenly lame. Having derived no benefit from the treatment of three leading doctors whom I consulted—after several months—I went to a famous surgeon, one of the best orthopædic men in England. His advice and treatment proved of no more use than that of the others I have mentioned. After this I was prepared to face a crippled existence, when a friend advised me to see Mr. Barker. Sharing the prejudices of most of my profession I hesitated to consult him, but finally, in sheer desperation, I did so. He was the first to diagnose that the cause of my lameness was a slipped semi-lunar cartilage. Under gas he reduced the dislocation and broke down the adhesions. The result was marvellous. At the end of about a fortnight I was able to walk with ease, and even to hop on the foot of the affected limb. The benefit received by myself and officer cousins when every other help had utterly failed has proved to me that Mr. Barker is possessed of

(*Continued on page* iv.)

